# Precarity and preparedness during the SARS‐CoV‐2 pandemic: A qualitative service evaluation of maternity healthcare professionals

**DOI:** 10.1111/aogs.14438

**Published:** 2022-08-11

**Authors:** Kaat De Backer, Jeremy M. Brown, Abigail Easter, Nina Khazaezadeh, Daghni Rajasingam, Jane Sandall, Laura A. Magee, Sergio A. Silverio

**Affiliations:** ^1^ Department of Women & Children's Health, School of Life Course & Population Sciences, Faculty of Life Sciences & Medicine King's College London London UK; ^2^ Medical School, Health Research Institute, Faculty of Health, Social Care & Medicine Edge Hill University Ormskirk UK; ^3^ Chief Midwifery Office, NHS England and Improvement London UK; ^4^ Maternity Services, St. Thomas' Hospital, Guy's and St. Thomas' NHS Foundation Trust London UK

**Keywords:** COVID‐19, health system shock, healthcare systems, maternity staff, precarity, pregnancy, preparedness, SARS‐CoV‐2

## Abstract

**Introduction:**

The SARS‐CoV‐2 pandemic has devastated populations, posing unprecedented challenges for healthcare services, staff and service‐users. In the UK, rapid reconfiguration of maternity healthcare service provision changed the landscape of antenatal, intrapartum and postnatal care. This study aimed to explore the experiences of maternity services staff who provided maternity care during the SARS‐CoV‐2 pandemic to inform future improvements in care.

**Material and methods:**

A qualitative interview service evaluation was undertaken at a single maternity service in an NHS Trust, South London. Respondents (*n* = 29) were recruited using a critical case purposeful sample of maternity services staff. Interviews were conducted using video‐conferencing software, and were transcribed and analyzed using Grounded Theory Analysis appropriate for cross‐disciplinary health research. The focus of analysis was on staff experiences of delivering maternity services and care during the SARS‐CoV‐2 pandemic.

**Results:**

A theory of “Precarity and Preparedness” was developed, comprising three main emergent themes: “Endemic precarity: A health system under pressure”; “A top‐down approach to managing the health system shock”; and “From un(der)‐prepared to future flourishing”.

**Conclusions:**

Maternity services in the UK were under significant strain and were inherently precarious. This was exacerbated by the SARS‐CoV‐2 pandemic, which saw further disruption to service provision, fragmentation of care and pre‐existing staff shortages. Positive changes are required to improve staff retention and team cohesion, and ensure patient‐centered care remains at the heart of maternity care.

## Graphical abstract

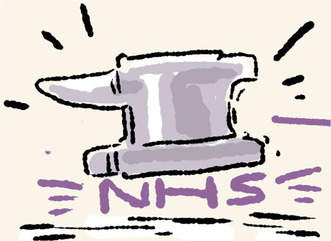

**Abbreviations**
GSTT: Guy's and St Thomas' NHS Foundation Trust
HCP: healthcare professional
NHS: National Health Service
SARS‐CoV‐2 or COVID‐19: The novel coronavirus pandemic

**Key message**

Maternity services are precarious, with many maternity professionals feeling their services are stretched. Retention‐related incentives and balance between service efficiency and patient‐centered care may help maternity staff to be better prepared for health system shocks in the future.

## 1 INTRODUCTION

The pandemic caused by the novel coronavirus, SARS‐CoV‐2 (COVID‐19), has been the first such an outbreak in a generation. The pandemic has devastated populations, and posed unprecedented challenges for healthcare services, staff and service‐users. In UK maternity services, rapid implementation of virtual care delivery (i.e. telehealth via video‐call or telephone), reduced face‐to‐face care, and limited birthplace options transformed the landscape of antenatal, intrapartum and postnatal care.^1^, ^2^

The rapid onset of the COVID‐19 pandemic caught healthcare systems around the world by surprise, leaving them uncertain about how they should, could, or would prepare for the challenges ahead.^3^, ^4^, ^5^ These included an unexpected surge in COVID‐19‐related hospital admissions^4^ and significant reductions in staff availability.^6^

Maternity services globally reported screening and containment of COVID‐19 in their workforce,^7^, ^8^ while facing high levels of staff burnout and negative mental health outcomes.^9^, ^10^, ^11^, ^12^ Staff shortages were caused by a myriad of SARS‐CoV‐2‐related reasons. Some staff “shielded” due to their own vulnerability to infection or that of household members^13^ while others self‐isolated following SARS‐CoV‐2 infection or that of close contacts.^14^ Some staff became more seriously ill, with particularly high numbers of minority ethnic healthcare professional (HCP) staff ultimately dying.^11^, ^15^, ^16^ In response, retired HCPs were encouraged to return to practice and part of the existing workforce was redeployed to frontline care of infected patients,^17^, ^18^ including re‐deployment of clinically trained non‐clinical staff (from managerial or research positions) to clinical roles, community‐based staff to hospital roles; and some maternity care staff to frontline clinical roles in emergency departments or COVID‐19 wards.^8^, ^19^, ^20^, ^21^ The result was inordinate strain upon healthcare systems, further fragmenting care.^22^

Whilst the concepts of burnout, understaffing and services running over capacity are not new to maternity services,^23^, ^24^, ^25^, ^26^, ^27^, ^28^ the circumstances of the pandemic exacerbated service‐level deficits. This study explored the system‐level response of reconfigured primary and referral maternity services in a large South London Trust providing care during the initial stages of the SARS‐CoV‐2 pandemic, with the aim of learning for future service delivery improvements.

## 2 MATERIAL AND METHODS

### 2.1 Patient and public involvement and engagement

This service evaluation was discussed with members of the National Institute for Health and Care Research (NIHR) Applied Research Collaboration (ARC) South London Patient and Public Involvement and Engagement (PPIE) meeting for Maternity and Perinatal Mental Health Research (July 2020), which has a focus on co‐morbidities, inequalities and maternal ethnicity; an NIHR ARC South London Work in Progress Meeting (October 2020), focusing on maternity and perinatal mental health research; a Maternity Services Directorate Briefing at Guy's and St Thomas' NHS Foundation Trust (GSTT) (January 2021), with a focus on health service improvements in safety and quality; an NIHR ARC South London Public Seminar (February 2021), which focused on COVID‐19 rapid response research; at two meetings of the Parent‐Infant coVid Organizational Academic Learning collaborative (PIVOT‐AL; November 2021, April 2022), which is leading the national response for policy makers during the pandemic; and to NHS England and Improvement's Chief Midwifery Office (December 2021), which focused on early insights from new research on maternity services to inform service COVID‐19 recovery. We received feedback on recruitment, study design and interpretation on findings from both lay and expert stakeholders, including members of the public, those with lived experience, health and social care professionals, researchers, and policy makers.

### 2.2 Design

Qualitative semi‐structured interviews were used to explore the experiences of service reconfiguration of HCPs at GSTT. Using a post‐positivist research paradigm, we adopted a critical realist ontology (enabling empathic understanding) and an objectivist epistemological stance (positioning interviewers and analysts as objective outsiders). This theoretical perspective was engaged in order to be faithful to our Grounded Theory methodology requirements and to our understanding that knowledge creation (i.e. interview narratives) can itself be falsified accounts of events but at the same time the “truths” or “lived realities” of a person; and it is the acquisition of knowledge—even false knowledge—which can bring us closer to understanding the true reality of a phenomenon. Respondents consented to interviews based on their understanding that their identity would not be disclosed to the Trust, and de‐identified data being published and shared with the Trust.

### 2.3 Respondent recruitment, setting, and data collection

Respondents (*n* = 29) were recruited between August and November 2020 at a time when the UK had imposed restrictions to daily life (including restricted numbers to both indoor and outdoor gatherings, and measures to reduce visitors to hospital patients to almost zero) in an attempt to reduce the spread of SARS‐CoV‐2. Initially, these were less stringent than the first UK lockdown (23 March–23 June 2020), but as infection cases increased again after the summer, a three‐tier system of local “lockdown” (Government‐mandated “stay‐at‐home” order) was announced in October 2020, with a second national “lockdown” coming into force in early November 2020. In parallel, maternity services continued with their restrictions which had been in force since March 2020, even during the summer of 2020, when people were encouraged to support the hospitality sector, through Government‐funded schemes.

We utilized a critical case purposeful sampling technique^29^ at GSTT, recruiting via directorate‐wide e‐mails inviting staff to take part in interviews. This enabled the Trust to act as the “critical case” and meant we attempted to achieve a maximum variation of respondents (e.g. professional roles) when recruiting from within the bounded setting of maternity services at one NHS Trust. To ensure anonymity from their clinical managers, interested respondents e‐mailed a non‐clinical member of the team (SAS), rather than a clinical colleague who circulated the recruitment e‐mails.

At the start of each interview recording, all respondents were asked to confirm their willingness to participate. Semi‐structured interviews^30^ following a chronological order, were conducted via video‐conferencing software,^31^ which allowed both flexibility of inquiry around a core set of questions and the interviews to take place during government‐mandated lockdowns and physical‐distancing restrictions (see Appendix S1 for Interview Schedule). Interviews were conducted by one of two authors (SAS—an academic Psychologist specializing in research on women's lifecourse health, who does not work clinically; KDB—a Perinatal Mental Health Midwife who at the time was working academically and clinically, but not at GSTT), dependent on availability. Interviews lasted an average of 50 minutes (range: 28–79 minutes) and were recorded and de‐identified while the audio was transcribed. Each transcript was given a unique number.

### 2.4 Data analysis

This analysis was focused on the system‐level response to service reconfiguration; analysis of individual‐level experiences will be reported elsewhere.

Grounded Theory Analysis^32^ appropriate for cross‐disciplinary health research^33^ was chosen and interviews were conducted until the point of theoretical saturation.^34^ Grounded Theory Analysis allows researchers to generate a theory from qualitative data which is focused on a specific population, experiencing a specific phenomenon, in a specific context. This theory can then act as a working hypothesis, and can be “tested” in subsequent studies by changing the population, phenomenon, or context, to see whether the theory holds true. This was assessed by employing “constant comparison”, where each transcript is coded and compared with previously analyzed transcripts and memo notes made by the researchers during the interviews and analysis, and “theoretical sampling”, where particular demographics of respondents may be associated with experiences divergent from the majority.^32^ By employing these established recruitment techniques, we were able to gain confidence in the selection of respondents to participate in interviews, increasing the overall trustworthiness of our data and subsequent analysis.

**FIGURE 1:**
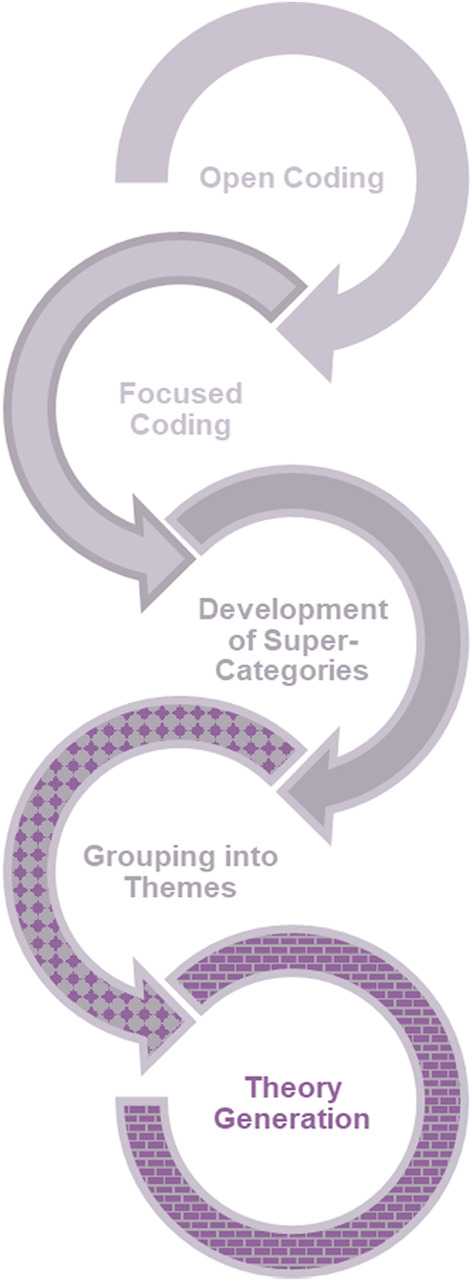
The Grounded Theory Analysis process, adapted from Silverio et al. (2019)^33^

Data were electronically coded, first “by hand”, using Microsoft Word, “line‐by‐line” or “open” codes (KDB) where each sentence of the data are coded with a key word from that sentence. Then data were subjected to a more nuanced, “focused” coding (SAS, KDB), which allowed open codes to be grouped more conceptually and these more conceptual codes to be applied to greater portions of the transcripts. Focused codes were analytically adapted and augmented to develop super‐categories (preliminary themes made up of groups of focus codes which are aligned or related), at which point a third analyst (JMB), masked to the original coding, checked for accuracy of super‐categories and reliability by re‐coding ~15% of transcripts.^33^ Finally, themes were developed by sorting and naming groups of super‐categories (see Figure 1).

The relationship between themes, which formed the basis of the grounded theory, was twice‐subjected to within‐team defense, to ensure that no other explanations were possible,^33^ and to allow refinement and ratification. Data are presented for each theme, with representative quotations provided in narrative prose, along with informative illustrations and figures.

### 2.5 Ethics statement

This project was approved as a service evaluation by Guy's and St Thomas' NHS Foundation Trust on July 7, 2020 (reference: 11046).

## 3 RESULTS

Respondents were multi‐ethnic, primarily female and, on average, in their mid‐40s. They were primarily midwifery (41%) or obstetrics staff (21%), with representation across a wide spectrum of care‐providers. Almost half were frontline clinicians, with about 40% of others in senior clinical or managerial roles. The vast majority were neither clinically vulnerable themselves nor had close family or household contacts who were. Most HCPs were experienced, with an average of more than 15 years' provision of clinical care and almost 10 years at their current Trust. A distinct minority (24%) were redeployed from their normal duties, and about two‐thirds had no history of confirmed or suspected COVID‐19 (see Table 1).

**TABLE 1:** Description of respondents

Characteristic	Respondents, *n* = 29 (%)
Professional background	
Midwifery	12 (41.4)
Obstetrics	6 (20.7)
Health visiting^a^	3 (10.3)
Other medical specializations (e.g. internal medicine)	2 (6.9)
Anesthesia	2 (6.9)
Neonatology	1 (3.4)
Nursing	1 (3.4)
Imaging sciences	1 (3.4)
Clerical	1 (3.4)
Maintenance/Cleaning/Security^b^	0 (0.0)
Position (level of seniority and/or primary responsibility)	
Frontline clinician (e.g. junior doctors, midwives)	14 (48.3)
Senior clinician (e.g. consultants)	4 (13.8)
Clinical manager (e.g. clinical staff responsible for delivery of a team)	4 (13.8)
Strategic leadership (e.g. clinical staff with senior management responsibilities)	4 (13.8)
Research (e.g. Clinically trained staff whose main role is to deliver clinical research)	2 (6.9)
Administrative (e.g. medical secretaries and office managers)	1 (3.4)
Maintenance/Cleaning/Security^b^ (e.g. service staff)	0 (0.0)
Years of experience (mean = 16.2 years)	
>5 years	3 (10.3)
6–10 years	7 (24.1)
11–20 years	10 (34.5)
21+ years	9 (31.0)
Years of experience at this trust (mean = 9.4 years)	
>5 years	10 (34.5)
6–10 years	10 (34.5)
11–20 years	9 (31.0)
21+ years	0 (0.0)
Redeployed^e^	
Yes	7 (24.1)
No	22 (75.9)
Age (mean = 44.6 years)	
18–24	0 (0.0)
25–34	5 (17.2)
35–44	10 (34.5)
45–54	10 (34.5)
55–64	4 (13.8)
≥65	0 (0.0)
Sex	
Female	26 (89.7)
Male	3 (10.3)
Ethnicity^c^	
White (White British, White Irish, White Gypsy/Traveler, White Other)	19 (65.5)
Black (Black African, Black Caribbean, Black Other)	5 (17.2)
Asian (Bangladeshi, Chinese, Indian, Pakistani, Asian Other)	3 (10.3)
Mixed (Mixed White/Asian, Mixed White/Black African, Mixed White/Black Caribbean, Mixed Other)	2 (6.9)
Other (Arab, any other)	0 (0.0)
Has had a positive COVID‐19 diagnosis^d^	
Yes	8 (27.6)
No	18 (62.1)
Possibly (unconfirmed)	3 (10.3)
Clinically vulnerable to COVID‐19	
Yes	2 (6.9)
No	27 (93.1)
Clinically vulnerable household or immediate family member	
Yes	4 (13.8)
No	25 (86.2)

^a^ In the UK, Health Visitors are nurses or midwives who have undertaken additional training in community public health nursing to become specialist community public health nurses.

^b^ Although recruitment was also open to members of staff from maintenance, cleaning, and security, we were unable to recruit any respondents from these aspects of the service.

^c^ Ethnicity was defined by respondents in response to the question: *“Could you tell me the ethnicity with which you identify?”,* and then grouped according to UK Government population statistics categories.

^d^ Respondents were recorded as “Possibly (Unconfirmed)” when they believed they had contracted COVID‐19, but never received clinical diagnosis.

^e^ Respondents were only deemed to have been redeployed when they had been asked to work in a clinical area where they had not previously worked as part of their contracted role at the Trust, or where their rotational working pattern had been completely re‐designed due to COVID‐19 service delivery reconfigurations.

**FIGURE 2:**
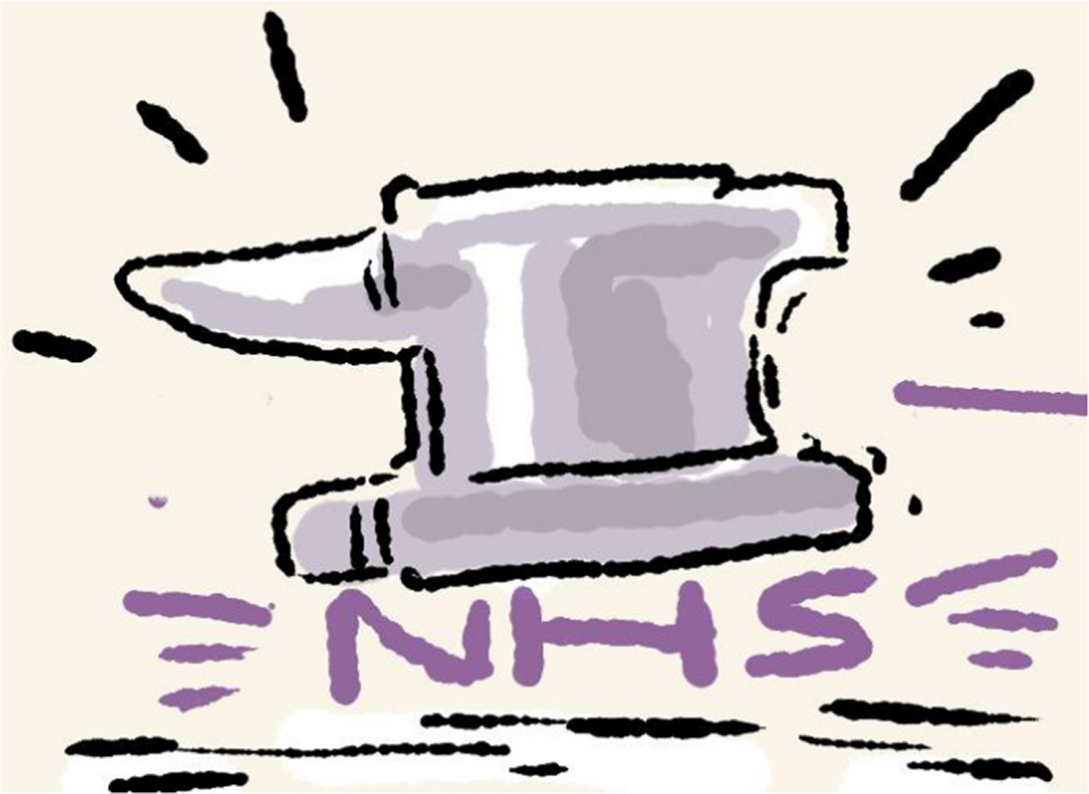
Illustration representing themes designed by a “live scriber” who drew this during a presentation of our data

The analysis comprised three main themes: “Endemic precarity: A health system under pressure”; “A top‐down approach to managing the health system shock”; and “From un(der)‐prepared to future flourishing”. Partial theoretical saturation was reached with 18 respondents, and full theoretical saturation achieved with 29. These themes are supported by the most illustrative quotations and a graphical representation (Figure 2), where appropriate. Additional quotations can be found in Table 2.

### 3.1 Endemic precarity: a health system under pressure

This theme captured descriptions of the maternity healthcare system pre‐pandemic, when respondents spoke about the health service strain that they had witnessed and experienced, and the service's obvious fragility:

It is a hard role to do within the hours that you have anyway, pre‐Covid. (Midwifery‐Clinical Manager)

I had to go part‐time in order to do that. Yes, I had to go part‐time because it was impossible to maintain a work‐life balance. (Midwifery‐Frontline Clinician)

Many respondents emphasized this was not due to insufficient training or competency of individual HCPs. Rather, there were structural issues within the healthcare system (at a macro‐level lens of understanding) which prevented staff from working efficiently and, on occasion, which set them up to fail:

…it's not a reflection or a criticism of healthcare professionals themselves, but just the system is not set up to support them to do their job properly… (Midwifery‐Frontline Clinician)

This was especially true with regard to communication and perceived bureaucracy, which HCPs felt prevented them from focusing on delivering high‐quality clinical work:

…working in the NHS… everything is a barrier, and everything takes too long, and we had huge issues with communication, team and bureaucracy about getting a project live – which still isn't up, despite them giving us the money to do it. That was painful, but that's just working in the NHS… (Midwifery‐Clinical Manager)

Furthermore, respondents mentioned being chronically understaffed, which was a fear for many as they could see the pandemic stretching across Europe towards the UK:

We had these meetings with the general managers where they were like, “What shall we do if it is 10% less staffing…20%, 30%, 40%, 50%?” I said to them, “Listen, we are 10% down all the time, so we know what to do when 10% of our staff are not there. That is every shift.” That was the lead‐up. (Midwifery‐Clinical Manager)

Respondents shared how understaffing often led to work not being done, as there were simply not enough staff:

I like being busy, and so it never really bothered me that it was a busy day. What did bother me and what was the thing that I didn't enjoy was then when it got too much, there was often not anyone around to help very much, so if you have to do 10 things in 5 minutes you had to do 10 things in 5 minutes and there often wasn't someone to lend a hand. Not necessarily because they didn't want to but just because they couldn't leave their woman. (Midwifery‐Frontline Clinician)

There was an emphasis on the longstanding issue with inadequate physical space, despite new recommendations for social distancing:

We've gone through years and years of cutting back on office space, cutting back on relaxation space and cutting back on [sigh]… everything really, to focus on trying to maximize our clinical space. (Neonatology‐Senior Clinician)

Respondents related this to how it made it difficult to plan the service delivery, when there were so many unknown variables on the horizon:

In the lead‐up there were lots of unknowns, lots of uncertainties. It was business as usual until we realized the scale of it, then it was a quick 10 days where we were like, we really need to prepare for the worst. What are we going to do? (Midwifery‐Clinical Manager)

…it was just… the uncertainty I found really, really challenging in that I didn't know whether or not in less than 2 weeks if I'd be working full‐time shifts or if I'd be working from home… (Midwifery‐Frontline Clinician)

Likewise, the emotional toll of the uncertainty began to show before the health system shock occurred, as staff braced for the expected effects to follow:

It is a lose‐lose situation being a manager and having your own life going on while there is a pandemic. You are trying to manage everybody else's emotions… (Midwifery‐Clinical Manager)

I worried a lot about was I going to be suddenly called to work in the Nightingale Hospital [an emergency hospital in London set up with the sole purpose to care for SARS‐CoV‐2 infected patients] when I'm a direct entry midwife, I've never worked in ICU, and I had a lot of anxiety around where was I going to end up working. (Midwifery‐Clinical Research)

Finally, more senior staff interviewed in this evaluation commented about the increased need for staff support and the dynamics among staff changing as the pandemic loomed:

I realized that very few people—even very seasoned, experienced people—were coping with the uncertainty and the pandemic. I was having to manage all of that. I felt that as someone who is okay with flexibility, volatility, and uncertainty. I had to try and provide some support for those around and above me… (Neonatology‐Senior Clinician)

### 3.2 A top‐down approach to managing the health system shock

Once the pandemic hit the UK, the health system experienced the shock of having to reconfigure with immeasurable speed and under less‐than‐ideal circumstances:

I think the first couple of months for us for survival… we were operating on a very basic level, but then again I don't know that there was very much that we could or would have done very differently. There were very limited options in what you could do in general. (Midwifery‐Frontline Clinician)

This rapidity of revisions to guidance which then had to be implemented was often reported negatively:

…things were rapidly changing. On a week‐to‐week basis, there were a lot of guidelines coming out that were changing, with regards to PPE, to protocol, to procedural things, that we had to keep up to date with. So eventually that was additional stress and fatigue added to normal work. There was obviously sick leave, colleagues getting sick or colleague's children coming down with fever and then they would need to self‐isolate. (Obstetrics‐Frontline Clinician)

Staff were particularly concerned when they felt that they had not been consulted or listened to when they raised concerns about the service reconfigurations that they were then expected to deliver:

I am not aware that they are consulting us very much at the moment about it all. It tends to be coming dictum from above. “This is what we are doing now.” (Midwifery‐Frontline Clinician)

I think they need to listen to the frontline staff more, because we have lots of ideas, we can create a service, but we need infrastructure and support to do that. And you need to allow us to do it but don't just leave us to do it either: there needs to be some structures. (Midwifery‐Clinical Manager)

This style of “command and control” over “engaged and responsive” leadership (at a structural or macro‐level and at a local or meso‐level lens of understanding) further disenfranchised staff, who felt their voices were not given due credence:

I feel like for God's sake, you've got to give us credit, we are the people on the frontline. I've got really helpful information, I see clients and I see them regularly and I feel like people… yes, I feel a bit dismissed sometimes. (Health Visiting‐Clinical Manager)

…this is what is being dictated from up high and we just have to make sense of it. So yes. And that's not very comfortable in our world. We do like to do things by consensus… (Health Visiting‐Strategic Leadership)

### 3.3 From un(der)‐prepared to future flourishing

Respondents continuously raised the need for preparedness for future pandemics and or other health system shocks, both locally at the Trust and nationally within maternity care:

I think there's been a lot of knee‐jerk approach instead of perhaps putting in place protective approaches… So that was frustrating… (Obstetrics‐Senior Clinician)

So, I think going forward we should be more prepared in terms of system preparedness and that means that if we need to expand on midwives, we should do—or the nurses in the community, we should do. The services in the community need to be maintained and be robust so that mothers don't necessarily need to come in until things are so acute, when it may be too late. (Obstetrics‐Senior Clinician)

A number of HCPs saw some positives in the pandemic, as an opportunity to reconfigure services to drive change:

We could give women a much better experience if things were more joined up and they weren't having to ring up and change appointments and not able to get through on the phone—all those things that were there pre‐Covid, but were accentuated when they weren't coming into the hospital at all… (Obstetrics‐Frontline Clinician)

…a lot of fat that was trimmed, essentially. And it was really good; it made things a lot more efficient; it rationalized care. It meant less wastage, I think. It demanded you to be more efficient in terms of planning the clinics. (Obstetrics‐Frontline Clinician)

Often these changes stemmed from the fact that opportunities for research and evidence‐based decision‐making had become more frequently discussed during the pandemic:

…the opportunities for research have been amazing and the will to get research sorted quickly and also present the findings associated with research has been amazing and I've learnt lots because we have been so on it, but if you don't provide time to hear about the research and think about the research, then it's pointless because it's got to be translated into improving clinical care. (Obstetrics‐Senior Clinician)

However, most were mindful that service reconfiguration, particularly when dramatic and swift, can be associated with crises of professional identity that have the potential to be empowering, however frightening at the time:

I know for the staff, for my team, it was quite unsettling because it was almost losing your own identity and trying to reinvent yourself and then trying to find out “Okay, who am I in this new role?” and then having to deal with the fear of “If I'm being deployed, am I going to be redeployed in an area that I feel confident or competent in working?” (Midwifery‐Strategic Leadership)

I think it started off by feeling really overwhelming with a huge amount of uncertainty and I would say genuinely some real fear of actually I might be put into an area where I don't feel confident and I don't want anything to happen to anyone that I'm looking after because I haven't done this for a long time, but actually in the end it felt relatively positive and in some ways it felt really good to be kind of more integrated with the overall hospital and team and to feel that we had contributed to something when things were difficult. (Midwifery‐Clinical Research)

**TABLE 2:** Supplementary quotations

Endemic precarity: A health system under pressure	A top‐down approach to managing the health system shock	From un(der)‐prepared to future flourishing
*I guess one of the really big things that felt like it changed on an emotional level rather than a practical level was it just suddenly felt that there was a huge level of uncertainty* (Midwifery‐Clinical Research) *What we had not thought about was the effect of men being in a vulnerable female space, the effect on women speaking to each other rather than just speaking to their partner, the effect on the staff of having all of these spare bodies around and trying to keep the place clean*. (Obstetrics‐Senior Clinician) *…it's very difficult to plan ahead because you do not really know what you are planning for*. (Midwifery‐Strategic Leadership)	*I think there were some of us that got pushed into places that we were… I think that was a bit hard. Also, because services had to change overnight, like my colleagues that cover Antenatal Clinic got told within a couple of days that they were moving to < Name of Hospital > for their clinic…* (Midwifery‐Frontline Clinician) *It felt like they came into our world and told us how to do something we had been doing, as far as we knew, perfectly well*. (Midwifery‐Frontline Clinician) *I think it was the way it was brought into us, being told that it was just going to happen straightaway, I think there was a little bit of a feeling of oh, well, is it not a worry for us being overheated and hot anymore?* (Midwifery‐Frontline Clinician) *…think it was just… the uncertainty I found really, really challenging in that I did not know whether or not in less than two weeks if I'd be working full‐time shifts or if I'd be working from home…* (Midwifery‐Frontline Clinician) *…it felt slightly tricky because we were not really sure what we were telling people and, in the beginning—and this is not a criticism at all because everybody obviously was dealing with this huge situation that developed out of nowhere and nobody knew what was happening—but I think it highlighted lots of, I guess, issues in the system…* (Midwifery‐Clinical Research) *I do not quite see why I cannot work from home. But there still seems to be a bit of… I do not know if taboo is the right word but just a lot of suspicion that if you are at home, you just do not really do very much, but actually I think I'm more productive at home. Yes. I guess it's the manager's call maybe, I do not know, or just the culture of the place…* (Midwifery‐Frontline Clinician) *So pretty much everything apart from gestational diabetes and Type 1, Type 2 in pregnancy disappeared. The preconception clinic was stopped. The rapid access clinic stopped. The community clinics closed. And many of the staff in diabetes were redeployed onto frontline, so there were about three of us left in the diabetes in pregnancy service and we basically ran it, with increasing numbers of pregnant women*. (Other Medical‐Frontline Clinician)	*…over time it felt better because the systems then were put in place and once people had asked those questions and you knew the answer then if people rang again, it made it easier*. (Midwifery‐Clinical Research) *I think the good thing about COVID is it's highlighted where some systems maybe needed to be improved and that got better over the course of the time…* (Midwifery‐Clinical Research) *Normally all the better birth stuff is trying to very much change care according to evidence and if suddenly lots of things happen that are not evidence‐based just because they had to happen, and they never got changed back that would feel like it's a shame*. (Midwifery‐Clinical Research) *I'm hoping that with research that's going on at the moment, things will not just go back to the way it was or they were but actually whoever is in charge and making big decisions will actually be looking at the research that's coming out and seeing what's good going forward as opposed to just assuming that pre‐COVID times was fine and let us just do that again*. (Midwifery‐Frontline Clinician) *But then things would change all the time so you would never sometimes know what on earth you were going to come into when you came into work. And I suppose then they did not know, like our managers did not know what would be happening, I do not know. So, sometimes it felt… It's quite unsettling when you do not know what you are going to be doing when you go to work…* (Midwifery‐Clinical Research)

## 4 DISCUSSION

Our Grounded Theory Analysis of 29 HCPs from a large South London Trust, illustrated three themes. “Endemic precarity” illustrated a maternity service under constant pressure pre‐pandemic—stretched and fragile, like other services within the NHS—due to the lack of built‐in “slack” to cope with additional strain. This pre‐existing precarity was drastically amplified by the SARS‐CoV‐2 pandemic. The "health system shock” theme described experiences of HCPs delivering care and fulfilling their professional roles within maternity care during the pandemic; the health system shock was unexpected and there was no “off‐the‐shelf” manual for how best to cope, and having continually to adapt and reconfigure services. Our final theme, focused on "un(der)‐preparedness and flourishing", demonstrating fractured and fragmented services, addressed the pervasive narratives that services (and staff) were under‐prepared at best, and un‐prepared at worst, to cope with the magnitude of the COVID‐19 health system shock.

**FIGURE 3:**
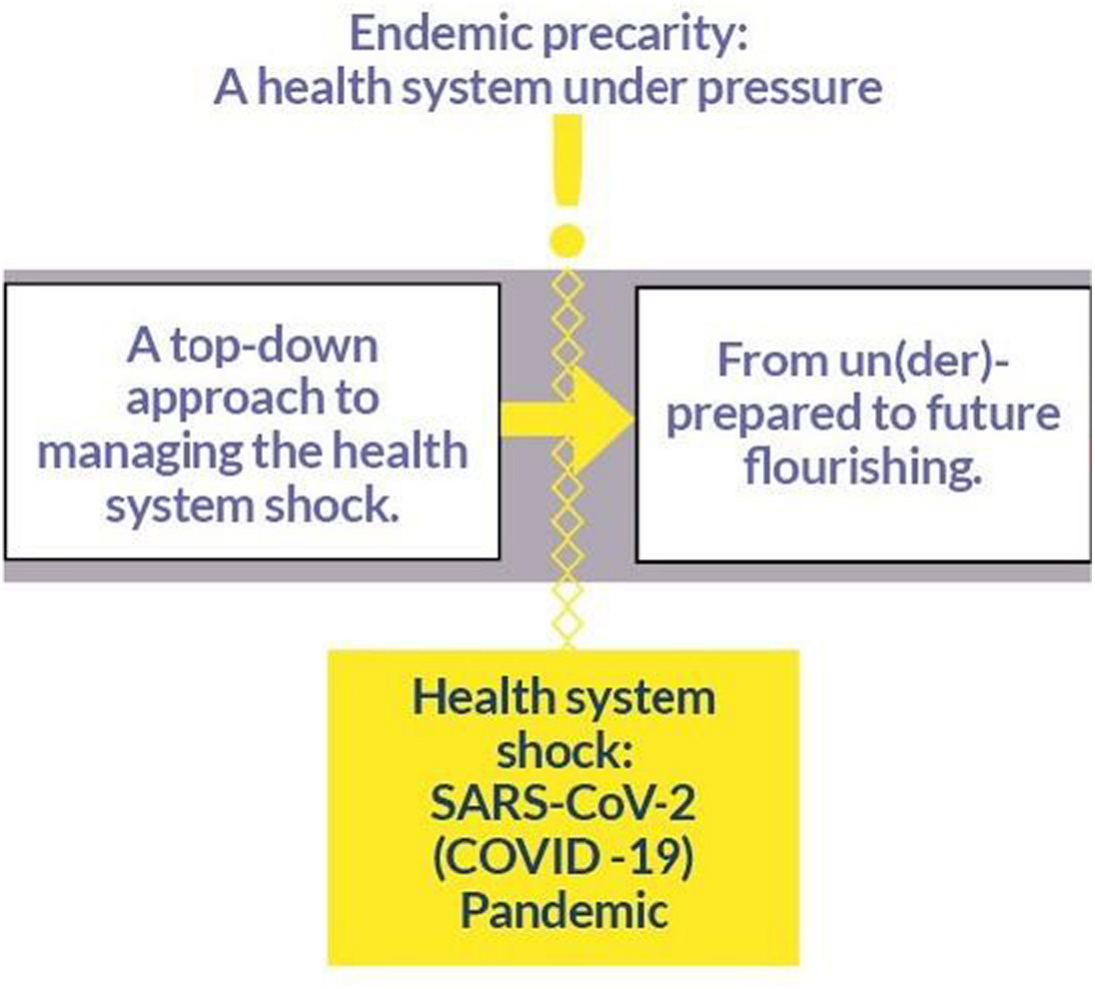
Representation of the Grounded Theory: Precarity and preparedness

Taken together—and in line with our Grounded Theory analytical approach—these themes can be interpreted as the theory: “Precarity and Preparedness” (Figure 3). In this theory, we see the specific population (maternity staff), phenomenon (delivering care during COVID‐19) and context (one NHS Trust in South London) struggle to prepare for and overcome the health system shock, because of the endemic precarity which already existed across and throughout the service. What we see, therefore, is a service reaction which stretches the expected in‐built resilience past its point of being plastic, rendering the service fractured, fragmented, and fragile. This has been seen globally in maternity HCPs.^35^, ^36^, ^37^ Ultimately, staff conceded that the service and they themselves were not prepared for this type of health system shock, or for the sustained level of added precarity it brought with it. The prolonged and cumulative effect of endemic precarity, and the un(der)‐prepared service, and the health system shock was occasionally seen as a chance to innovate and transform,^38^ albeit usually with a top‐down or “command and control” style approach, which was not always appraised positively. Furthermore, innovation has often been reported as a proxy term for the reality of time being spent on paring back services and delivering only essential care causing poorer outcomes for women, their families, and their babies;^2^, ^39^, ^40^, ^41^, ^42^, ^43^, ^44^ and demoralizing staff who did not believe they were providing the level of care they ought to and were trained to deliver.

To address both previous concerns about over‐stretched maternity services,^23^, ^24^, ^25^, ^26^, ^27^, ^28^ as well as the current challenges after the pandemic health system shock, we have identified domains suggesting that shifts in maternity services are required to allow its workforce to work sustainably and efficiently.

First, incentives to retain healthcare staff are long overdue, as evidenced by staff narratives during the pandemic. Maternity services have functioned with increased demand and reduced staffing resource and capacity for too long. Additional stressors to workforce retention, such as the end of “freedom of movement” of trained HCPs from the European Union due to “Brexit”, have exacerbated shortages of staff, and those who have stayed, are having to cope with unrealistic work pressures. Incentives to retain them, as well as attracting new healthcare staff and increasing healthcare students' cohorts, are required to ensure workloads are realistic, manageable, and sustainable.

Secondly, to restore the reputation of the NHS and instill a sense of identity among its staff, a balance needs to be struck between making services efficient, while maintaining a patient‐centered focus. Relatively “simple” changes which proved possible during the COVID‐19 pandemic, such as hosting multiple clinics during the same prenatal visit, offering the choice of virtual care appointments, and allowing women more flexible access to care, created opportunities to achieve new ways of delivering high‐quality care. Continued efforts are needed to further galvanize these opportunities, well beyond the initial health system shock.

This study formed part of a rapid research response to the SARS‐CoV‐2 health system shock and, as such, captures the early perceptions of delivering care during the pandemic by maternity staff at one NHS Trust. While the focus on one Trust may be conceived of as a limitation, the breadth of professional roles recruited to interviews makes our findings more generalizable. Our sample was fairly representative of the clinical staff within the Trust, but may not represent the whole body of staff in terms of ethnicity and gender when non‐clinical, service and maintenance staff who work at GSTT are considered more widely.^45^ We accept that experiences of maternity care service delivery may differ within even a single Trust, but we believe our findings could be generalized to similar services in other Trusts, or indeed in other high‐resource settings where healthcare is provided free at point‐of‐access, nationally, to all those who require care. Further strengths lie in the recruitment of respondents from a wide range of ethnicities, age, years of experience, and seniority, although we appreciate that the gender‐split in this study could be identified as problematic (though reflective of maternity care more broadly). A further strength lies in the fact that the researchers who undertook the interviews and carried out the analysis were independent of the Trust and so did not have preconceived biases about its working practices, and the wider research team involved those not working from the Trust, those who were working or had worked for the Trust, and one member who was not based in London who acted as a “critical friend” for the study group.

Future research will be able to take the theory developed in this analysis, and by changing the specific population, phenomenon, or context, will be able to “test” whether the theory holds true.^33^, ^46^ Our theory can also be re‐tested at the same Trust in due time, to re‐visit the themes emerging from this study and assess progress or positive changes made since.

## 5 CONCLUSION

Maternity services were under significant strain before the outbreak of the COVID‐19 pandemic, leading to an inherently precarious healthcare system. This precarity was subsequently exacerbated by the health system shock, as SARS‐CoV‐2 caused disruption to staff availability and service delivery, with enduring consequences leading to fragmentation of care and systemic staff shortages. Positive change is required to improve staff retention and balance service efficiency while sustaining high quality, patient‐centered care at the heart of the NHS.

## AUTHOR CONTRIBUTIONS

Conceptualization: SAS, JS, LAM, DR, AE. Methodology: SAS. Software: SAS; KDB, JMB. Validation: SAS, JMB, LAM, DR, NK. Formal analysis: SAS, KDB. Investigation: SAS, KDB, JMB. Resources: SAS, DR, JS, LAM. Data curation: KDB, SAS. Writing—Original Draft: SAS, KDB. Writing—Review & Editing: LAM, DR, NK, JMB, AE, JS. Visualization: SAS, KDB, DR. Supervision: JS, LAM. Project administration: SAS. Funding acquisition: LAM, SAS, AE, JS.

## FUNDING INFORMATION

King's College London King's Together Rapid COVID‐19 Call, successfully awarded to LAM, SAS, AE & colleagues (reference: 204823/Z/16/Z), as part of a rapid response call for research proposals. The King's Together Fund is a Wellcome Trust funded initiative. NIHR Senior Investigator Award held by JS (reference: NIHR200306).

## CONFLICT OF INTEREST

SAS, KDB, AE, and JS (King's College London) are currently supported by the National Institute for Health Research Applied Research Collaboration South London (NIHR ARC South London) at King's College Hospital NHS Foundation Trust. JS (King's College London) is an NIHR Senior Investigator. SAS (King's College London) is in receipt of a Personal Doctoral Fellowship from the NIHR ARC South London Capacity Building Theme and is also supported by the NIHR Senior Investigator Award of JS. KDB (King's College London) was previously supported by the National Institute for Health Research Applied Research Collaboration East of England (NIHR ARC East of England) at Cambridgeshire and Peterborough NHS Foundation Trust. The views expressed are those of the authors and not necessarily those of the NIHR or the Department of Health and Social Care.

## Supporting information


Appendix S1
