# Supplementary material for: Precarity and preparedness during the SARS‐CoV‐2 pandemic: A qualitative service evaluation of maternity healthcare professionals
Source: Acta Obstet Gynecol Scand. 2022 Aug 11:10.1111/aogs.14438. Online ahead of print. doi: 10.1111/aogs.14438 (PMC9538337; doi:10.1111/aogs.14438)
Supplement: Supplementary file 1 — Appendix S1 [file AOGS-9999-0-s001.pdf]

Gathering maternity healthcare professionals' (HCP) experiences during COVID-19 Pandemic and views on service recovery.

Thank you for taking the time to be interviewed in relation to the HCP's Experiences of Maternity Care During COVID-19 study.

Introductions: You have been delivering maternity care. You are one of a number of HCPs who said that they'd be happy to talk to us some more – so thank you! What I'd like to do in this interview is to ask you in a bit more details about your experiences of the maternity care you have delivered, and for you to tell me, in your own words, what happened during the COVID-19 pandemic, including any changes you might have experienced. We also want to know your views on service recovery to improve quality and safety of services.

There are no right or wrong answers – we are interested in the full range of your experiences and the opinion of the women you have been looking after. The interview will take approximately 30 mins–1hr.

Do you have any questions before we begin? If not, I shall now start to record.

INFORMATION CHECKING:

As we begin, and before we talk in detail about your experiences, may I check that the information I have is correct – about the you and the hospital in which you work?

- **Hospital**: *Check we have correct information.*
  - Main hospital base
  - Began working there?
- **General background information:** Also, can I have some background information about you.
  - Age
  - Ethnicity
  - Current role
  - How long have you been qualified as a [Professional Title].
  - Were you a high risk group who required shielding/self-isolation? Was anyone within your immediate family/household in the high risk group and how did that impact on you?
  - Did you at any point have COVID?

WORK-LIFE BEFORE COVID-19:

Now I would like to ask you a bit more about your role in delivering maternity care. Could you tell me what your role consisted of before COVID-19? *[Let interviewee volunteer as much information as possible before probing.]*

- Probes
  - Day to Day?
  - Any special interests/clinics?
  - What was enjoyable and less enjoyable about your job?
  - How did you manage a work-life balance?

CHANGES IN WORK-LIFE DUE TO COVID:

Now I would like to ask you a bit more about how this might have changed. Could you tell me about what COVID-19 has meant for your job? *[Let interviewee volunteer as much information as possible before probing.]*

- Probes
  - Key changes?
  - Day to Day changes?
  - Logistical changes? (i.e. stepping into a clinical role, when not usually clinical, remote working due to shielding or at high risk group)
  - Changes to shift pattern?

KING'S College LONDON

- Were you able to have input and raise your concerns during the reconfiguration/process?
- Did you receive support in terms of IT/training/upskilling during this transition?
- Were you able to suggest making changes and were these taken seriously?
- Impact on commute? Did you have to change where you lived?
- Impact on family time?

My next questions are specifically related to the delivery of virtual care. What have your experiences of this been like?

- Probes
  - Changes to services, continuity of care, home birth and alongside unit, specialist clinics, scans, postnatal care
  - Telephone consultations/Video conferences?
  - How did this affect workload?
  - Were there any technological challenges?
  - How was it when the woman's' first language wasn't English?
  - Any fears or concerns about patients (not specific patients, but groups e.g. DV etc.)

How do you perceive this has affected the rest of your colleagues?

- Probes
  - How has this made them feel?
  - Team dynamics?
  - Upskilling/team learning?
  - Have you felt pressured to work in environments when you have not felt safe?

For the second half of the interview I would like to ask you about how the COVID-19 pandemic may have affected the maternity care you deliver to women. Would you like to take a break, or are we okay to continue?

What have these changes meant for the women in your care? *[Let interviewee volunteer as much information as possible before probing.]*

- Probes
  - Anything detrimental?
  - Anything better than before?
  - What about their partners?
  - Biggest difficulty and biggest success?

How have you perceived women's acceptance and understanding of the changes you have had to make? *[Let interviewee volunteer as much information as possible before probing.]*

- Probes
  - Have there been any challenges?
  - How have you overcome any challenges?

How would you compare the maternity care offered before and during the 'lockdown'?

- Probes
  - What are the positive /negative aspects in your view in relation to safety?
  - What are the positive/negative aspects in your view in relation to women's experience?
  - Are there any aspects that you would like to embed in the service going forward?
  - Safeguarding issues – did they feel there was an increase or were they aware that there may have been an increase?

And what have your personal experiences been like? How has this made you feel?

- Probes
  - What has been most challenging? If any challenges are identified, have you felt able to raise those concerns with your colleagues or line manager? Have your concerns been heard and acted on?
  - Has anything new been fun?
  - What has been the best and worst experience since the changes?

- Has the changes impacted on your family life, e.g. your living arrangements, impact on children or partner?
- Have you had physical space to do virtual consultations or have these been working from home?
- Have you changed your lifestyle behaviour during this time (both positively and negatively)?
- Emotional impact? Physical impact? Fear? Isolation?

LOOKING FORWARD TOWARDS MATERNITY CARE AFTER COVID-19:
As I am sure you will know, particular groups have been identified as at higher risk to COVID-19 than others, such as those members of the BAME communities. This has led to some people delaying health seeking behaviours, due to fear of diagnosis, stigma of having the virus, and their increased risk of death. Do you believe the Trust has put in sufficient measures to mitigate against this? And this can be in terms of encouraging and protecting both women in your care, and staff members.

- Probes:
  - How would a culturally sensitive education sessions/ risk assessment tool address this?
  - What support should be given to BAME and people with pre-existing conditions?
  - What approach would mitigate such fears and encourage staff to speak out?

My final questions are about you reflecting on what maternity care might look like throughout the rest of the pandemic and after the pandemic is over. What do you think will happen next? *[Let interviewee volunteer as much information as possible before probing.]*

- Probes
  - How much of this do you feel is in your control?
  - Is there any preparation going on now to mitigate any future changes?
  - Any thoughts or feelings on these adaptations?

What would you like to happen after the pandemic?

- Probes
  - What things will/should go back to normal? Why?
  - What is new that will/should be implemented? Why?
  - Will any key changes be kept? Why?
  - Do you believe these opinions differ from that of the women in your care?

What advice would you give to women who are having to use maternity services during the pandemic?

- Probes
  - Do you believe they have accepted the changes to routine care well?
  - Do you believe that the changes you mentioned before would be acceptable to women in a post-pandemic time?

Do you have any advice for HCPs in a similar situation to yourself?

- Probes
  - What has been your major point of learning?
  - Any other reflections?

What would you do differently in a second wave of this pandemic or a pandemic in the future?

I have come to the end of my interview questions, but is there anything at all that you would like to add that perhaps I haven't asked you about? Or do you have any questions for me?

Thank you very much for your time.
